# Alginate-modified mesoporous bioactive glass and its drug delivery, bioactivity, and osteogenic properties

**DOI:** 10.3389/fbioe.2022.994925

**Published:** 2022-10-05

**Authors:** Haiyan Yao, Jun Luo, Yunyun Deng, Zhihua Li, Junchao Wei

**Affiliations:** ^1^ School of Stomatology, Nanchang University, Nanchang, China; ^2^ College of Chemistry, Nanchang University, Nanchang, China; ^3^ Jiangxi Province Key Laboratory of Oral Biomedicine, Nanchang, China; ^4^ Jiangxi Province Clinical Research Center for Oral Disease, Nanchang, China

**Keywords:** mesoporous bioactive glass, surface modification, alginate, drug loading, bone regeneration

## Abstract

Mesoporous bioactive glass (MBG) is widely used in bone tissue repairing and drug loading. However, burst release of drug and poor compatibility with other materials limited its application. It is an effective way to modify MBG with a polymer brush to improve the properties. Herein, an alginate-modified MBG was prepared, and then, the effects of ALG on the properties of MBG were investigated. The results demonstrate that ALG could improve the drug loading efficiency, prolong drug release times, and make orderly deposition of apatite on the surface of MBG. Furthermore, MBG@ALG significantly promoted the osteogenic differentiation of MC3T3-E1 cells, demonstrating that surface modification of MBG by ALG can improve its properties, which will further broaden the application of MBG in tissue engineering.

## 1 Introduction

Bioactive glass (BG) is widely used in bone tissue engineering scaffold (Vallet-Regi and Salinas, 2021) and polymer composites for bone regeneration (Zhao et al., 2021) due to its excellent biocompatibility and osteogenic properties. Mesoporous bioactive glass (MBG) not only possesses the advantages of BG but also showed superior *in vitro* bone-forming bioactivities (Rahaman et al., 2011; Schumacher et al., 2021), high porosity, and specific surface area. Therefore, MBG has been widely used as a drug delivery system (Lopez-Noriega et al., 2010) for the treatment of bone tissue diseases; for example, various functional molecules, such as small molecule drugs (alendronate) (Ravanbakhsh et al., 2019) and growth factors (BMP-2, VEGF) have been loaded into MBG (Kim et al., 2016; Schumacher et al., 2017). However, the poor binding ability with other materials (Kargozar et al., 2019), burst release of drugs, and easy formation of protein crowns in biological media (Pontremoli et al., 2020; Sharifi et al., 2022) have limited the application of MBG. Therefore, it is of much importance to tailor the properties of MBG to improve its applications.

The surface of nanomaterials play critical roles in many physical and chemical processes, while the surface ligands or molecules binding to the surface are essential components of nanomaterials and affect its interactions with other materials or biological systems (Kango et al., 2013; Boles et al., 2016), and thus many works have been carried out to tune the surface of nanomaterials with organic ligands and polymer brushes (Boles et al., 2016; Heinz et al., 2017). As for MBG, the surface molecules or polymer brushes may work as gatekeepers and tune its drug delivery ability, for example, various molecules, such as 3-aminopropyltrimethoxysilane (Wang et al., 2018) and poly-L-glutamic acid (Das et al., 2021) have been used to enhance its drug loading efficiency and prolong the drug release time. In addition, the surface polymer brushes may have great effect on the bioactivity of MBG (Kargozar et al., 2019), which will greatly affect the biomineralization of the apatite and the binding ability of MBG to tissues. For example, poly (amidoamine) (PAMAM) dendrimer-coated mesoporous bioactive glass nanoparticles (MBG) (PAMAM@MBG) were prepared and used for treatment of dentine hypersensitivity (Bae et al., 2019), while the results demonstrated that the PAMAM@MBG had excellent mineralization ability and showed a better occluding effect for dentinal tubules than that of MBG. In addition, the surface modification of nanomaterials improves the phase compatibility between nanomaterials and polymer matrix, and thus improves the mechanical properties of polymer composites (Kango et al., 2013). For instance, polydopamine has been coated on the surface of MBG, which greatly improves the mechanical properties of MBG/PLLA scaffolds (Xu et al., 2018). Generally, it has been an effective method to graft polymers or organic molecules on the surface of MBG to improve its charming properties. However, it is still a great challenge to modify the surface of MBG with mild conditions and improve multiple performances of MBG simultaneously.

Alginate (ALG) is an anionic polymer with good biocompatibility and low toxicity, and has been widely applied in biomedical applications (Lee and Moone, 2012). ALG can be tethered on the surface of nanomaterials to improve the colloidal stability of various nanobuilding blocks. For example, ALG-modified SiO_2_ (Yan et al., 2019), carbon nanotubes (Yao et al., 2021), and upconversion nanoparticles (Cao et al., 2020) show good colloidal stability and biocompatibility. Furthermore, the ALG molecules can also reduce protein adsorption to nanomaterials and inhibit the formation of protein crowns. Mooney’s group prepared cysteine-functionalized ALG--derived polymers as stabilizers to coat the surface of gold nanoparticles (GNPs), which increase the stability of the GNPs and reduce the adsorption of proteins on GNPs (Kodiyan et al., 2012). In addition, ALG has immunomodulatory effect, which can accelerate the wound healing by promoting the anti-inflammatory polarization of macrophages (Bygd and Bratlie, 2016; Kerschenmeyer et al., 2017). In addition, the ALG polymers contain multiple reactive groups, such as hydroxyl and carboxyl groups, which could be an ideal candidate for the further functionalization of materials with special functions, for example, cell targeting ligands and therapeutic drugs (Ahmad Raus et al., 2021). Therefore, it is definite that ALG as a polymer brush can greatly alter the biological properties of nanomaterials. Herein, if ALG was combined with MBG, the advantages of both the materials may have synergistic effect and realize the improvements of the multifunction of MBG.

**FIGURE 1:**
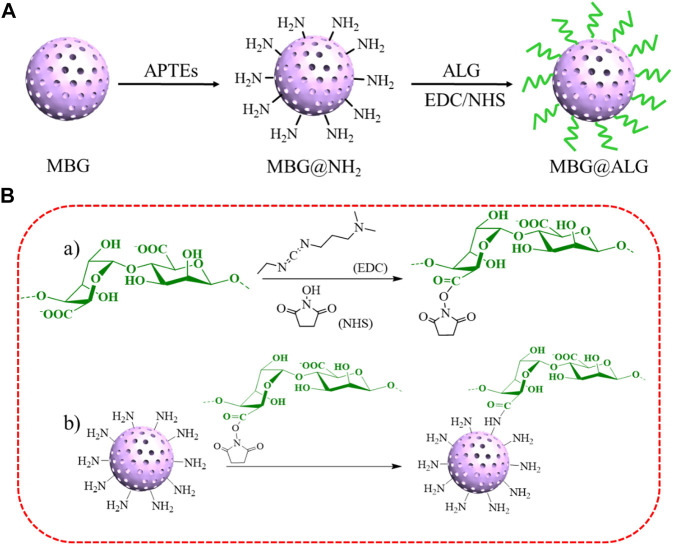
Schematic illustration of the construction of MBG@ALG **(A)** and reaction scheme **(B)**.

In this work, ALG was grafted on MBG (MBG@ALG) *via* a simple method (Figure 1), and all the reactions were conducted at mild temperature and no toxic solvents were used. In addition, the method is universal and can be used to modify the surface of other kinds of nanomaterials and improve their properties. In addition, the effect of ALG on drug loading efficiency, bioactivity, biocompatibility, and osteogenic properties of MBG were investigated. The results demonstrated that ALG brush can enhance drug loading efficiency, prolong drug release time, promote the orderly deposition of apatite, and improve osteogenic performance of MBG, having a positive effect to improve the performance of MBG and broaden its application in bone tissue engineering.

## 2 Materials and methods

### 2.1 Materials

Hexadecyl trimethyl ammonium bromide (CTAB, 98%), tetraethyl orthosilicate (TEOS), triethyl phosphate (TEP), calcium nitrate tetrahydrate (Ca(NO_3_)_2_.4H_2_O), ammonium hydroxide (NH_3_.H_2_O), and ethanol were purchased from Sinopharm Chemical Reagent Co., Ltd. (Shanghai, China.). Sodium alginate (ALG, the viscosity of ALG (1%, Brookfield LV, 20°C) is 350–550 cP), 3-aminopropyltrimethoxysilane (APTES), ethyl-3-(3-dimethylaminopropyl) carbodiimide hydrochloride (EDC), N-hydroxysuccinimide (NHS, 98%), and sodium alginate (98%) were purchased from Aladdin Scientific Co., Ltd. (Shanghai, China). Acridine orange/ethidium bromide was purchased from BestBio Co., Ltd. (Shanghai, China). The MTT kit and Alkaline Phosphatase Color Development Kit were purchased from Beyotime Co., Ltd. (Shanghai, China). Alizarin Red S (ARS) staining was purchased from Solarbio Co., Ltd. (Beijing, China).

### 2.2 Methods

#### 2.2.1 Preparation of MBG@ALG

First, MBG was synthesized by a reported method (Ravanbakhsh et al., 2019). Briefly, 0.7 g of CTAB was dissolved in 33 ml of water under stirring, and then 10 ml of ethyl acetate was added. 30 min later, 7 ml of aqueous ammonia (3 mol. L^−1^) was added. 15 min later, 3.6 ml of TEOS, 0.36 ml of TEP, and 2.277 g of Ca(NO_3_)_2_.4H_2_O were sequentially added to the abovementioned mixture at every 30 min interval. The solution was vigorously stirred for another 4 h, the precipitate was collected by centrifugation, and then washed three times with ethanol and water in turn. The raw product was dried at 60°C for 24 h and then calcined at 650°C for 6 h to obtain MBG.

Second, 0.3 g of MBG and 0.6 ml of APTES were added in 100 ml of ethanol, and then the mixed solution was refluxed at 80°C for 24 h. The precipitate was collected by centrifugation, washed three times with ultrapure water, and freeze-dried to obtain MBG@NH_2_.

Third, 0.25 g of ALG was dissolved in 50 ml of water and stirred for 24 h, then 0.2399 g of EDC and 0.1439 g of NHS were added in the solution and stirred for 2 h. 0.25 g of MBG@NH_2_ was added in the abovementioned solution and stirred for 24 h and then centrifuged and washed with deionized water and lyophilized.

#### 2.2.2 Characterization

The weight loss of different samples was measured by using a thermal gravimetric analyzer (TGA, Perkin Elmer, TGA 4000, United States). The crystalline structure was measured on X-ray diffraction (XRD, SmartLab 9 KW, Japan). The morphology of different samples was observed using a transmission electron microscope (TEM, JEOL, JEM-2100, Japan) and scanning electron microscope (SEM, Thermo Fisher Scientific, Apero C HiVac, United States).

#### 2.2.3 Drug loading and release

Simvastatin (SIM) was used as a model drug. Briefly, 10 mg of MBG and MBG@ALG was dispersed in 10 ml of ethanol, and 5 mg of SIM was added and stirred for 24 h, respectively. Then, the supernatant was centrifuged and the absorbance value at 238 nm of the supernatant was measured. The mass of SIM in the supernatant was calculated using the measured SIM standard curve. The loading efficiency (%) and drug content (%) were calculated using the following formula:

Loading efficiency (%)=(weight of drug in MBG/initial weight of drug)×100%,

Drug content (%)=(weight of drug in MBG/weight of MBG)×100%.

*in vitro* drug release of SIM from MBG and MBG@ALG was performed by the dialysis bag diffusion method. 2 mg of MBG and MBG@ALG loaded with SIM was suspended in 2 ml of phosphate-buffered saline (PBS, pH7.4) and introduced into the dialysis bag. The dialysis bag was kept at 30 ml of PBS containing 20% ethanol and transferred to constant temperature shaker. At each time point (1, 3, 12, 24, 24, 48, and 96 h), 2 ml of the release medium was taken out and replaced with equal amounts of fresh medium. The amount of released SIM was evaluated by ultraviolet-visible (UV) analysis at a wavelength of 238 nm.

#### 2.2.4 Bioactivity test in simulated body fluids

An SBF (simulated body fluid) solution was first prepared according a reported method (Kokubo and Takadama, 2006), and then 20 mg of MBG and MBG@ALG powder were soaked in 20 ml of SBF at 37°C for 7 days in a shaking incubator. The SBF was refreshed every 24 h. Then, SEM was conducted to evaluate apatite formation.

#### 2.2.5 Biocompatibility test

The samples were irradiated under UV for 2 h, and then added into a culture medium. The concentration was diluted with a culture medium to 500, 250, 125, 50, and 25 μg/ml 100 μl of the culture medium containing MC3T3-E1 cells at a density of 8 × 10^4^ cells/mL were seeded into a 96-well plate and incubated for 24 h for cell attachment. Subsequently, the culture media was replaced with 100 μl of materials at concentrations of 500, 250, 125, 50, and 25 μg/ml. After the cells were coincubated with nanoparticles for 24 h, the medium was sucked out and PBS was added for washing several times, then the MTT reagent (10 μl) mixed with 100 μl of fresh medium was added to each well. After 4 h of incubation, the medium was removed and 150 μl of DMSO was added to fully dissolve the formazan, and then the OD value at 490 nm was measured.

AO/EB (Acridine Orange/Ethidium Brmide) staining was used to further detect the apoptosis of MC3T3-E1 cells. 100 μl of culture media containing MC3T3-E1 cells at a density of 8 × 10^4^ cells/mL were seeded into a 96 plate and incubated at 37°C under atmosphere of 5% CO_2_ for 24 h. Subsequently, the culture media was replaced with 100 μl of fresh medium containing samples at various concentrations of 500, 250, 125 μg/ml. After 24 h co-incubation, fresh medium was used to wash each well several times, and then AO/EB reagent was added to each well for 30 s. Finally, the survival of cells was observed by fluorescence microscopy.

#### 2.2.6 Osteogenesis differentiation

100 μl of culture media containing MC3T3-E1 cells at a density of 1 × 10^4^ cells/mL were seeded into a 96-well plate and incubated at 37°C under atmosphere of 5% CO_2_ for 24 h. Subsequently, the culture media was replaced with 100 μl of medium containing samples at concentrations of 250 μg/ml. After 7 and 14 days, PBS was used to wash the plate several times, and the cells were fixed with 4% paraformaldehyde for 30 min. Then, the cells were stained with Alkaline Phosphatase (ALP) Color Development Kit and Alizarin Red S (ARS) Staining.

## 3 Results and discussion

### 3.1 Characterization of different samples

**FIGURE 2:**
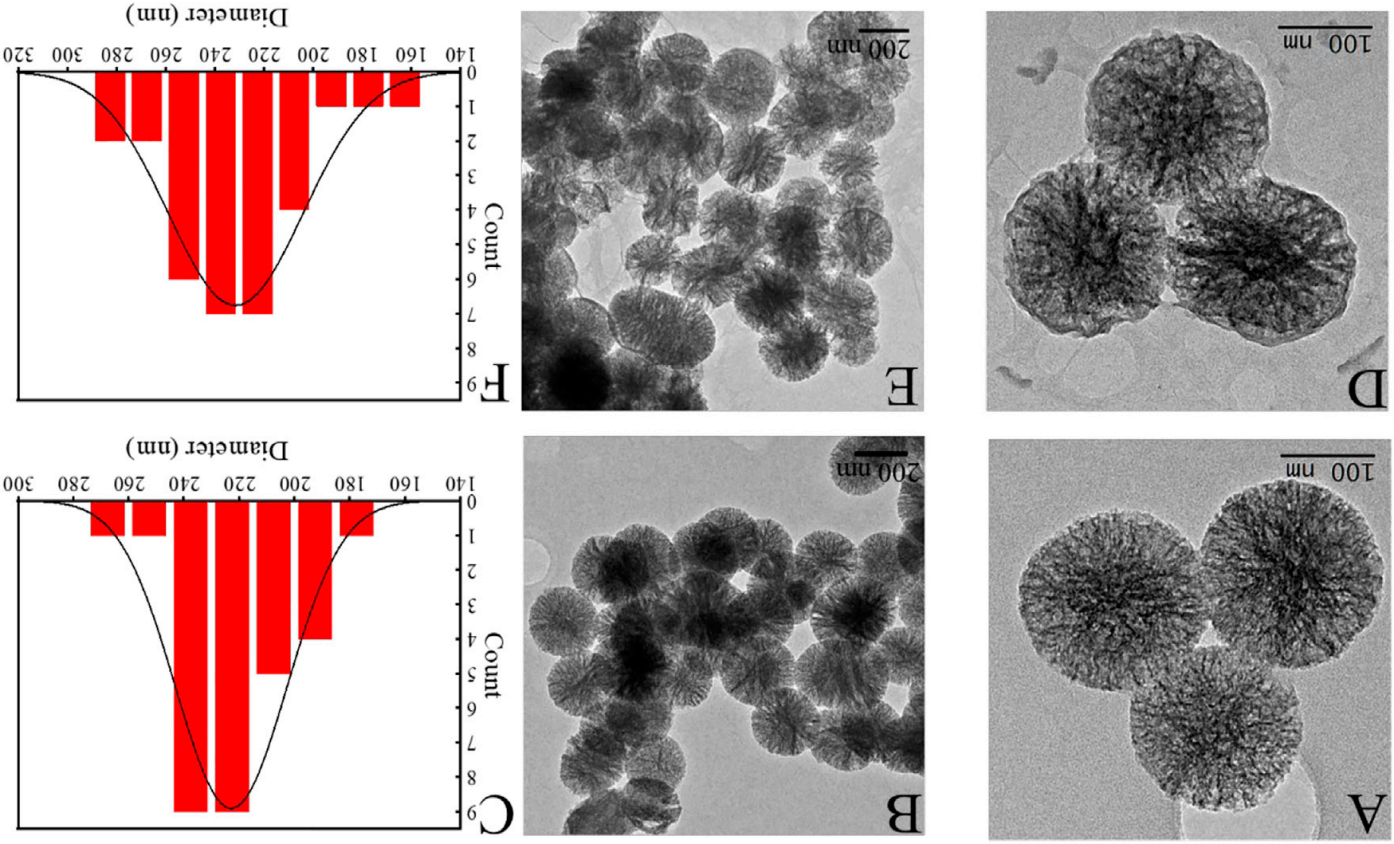
TEM images of MBG **(A,B)** and MBG@ALG **(D,E)**. Particle size analysis of MBG **(C)** and MBG@ALG **(F)**.

MBG was spherical, mono-dispersed, and porous particles (Figures 2A,B), the diameter of MBG was 222 ± 19 nm (Figure 2C). When ALG was grafted on the surface of MBG, a polymer coating could be clearly observed on the surface of MBG (Figures 2D,E), and the mean diameter of the MBG@ALG was 232 ± 22 nm (Figure 2F), which demonstrated that ALG was successfully grafted onto the MBG surface.

**FIGURE 3:**
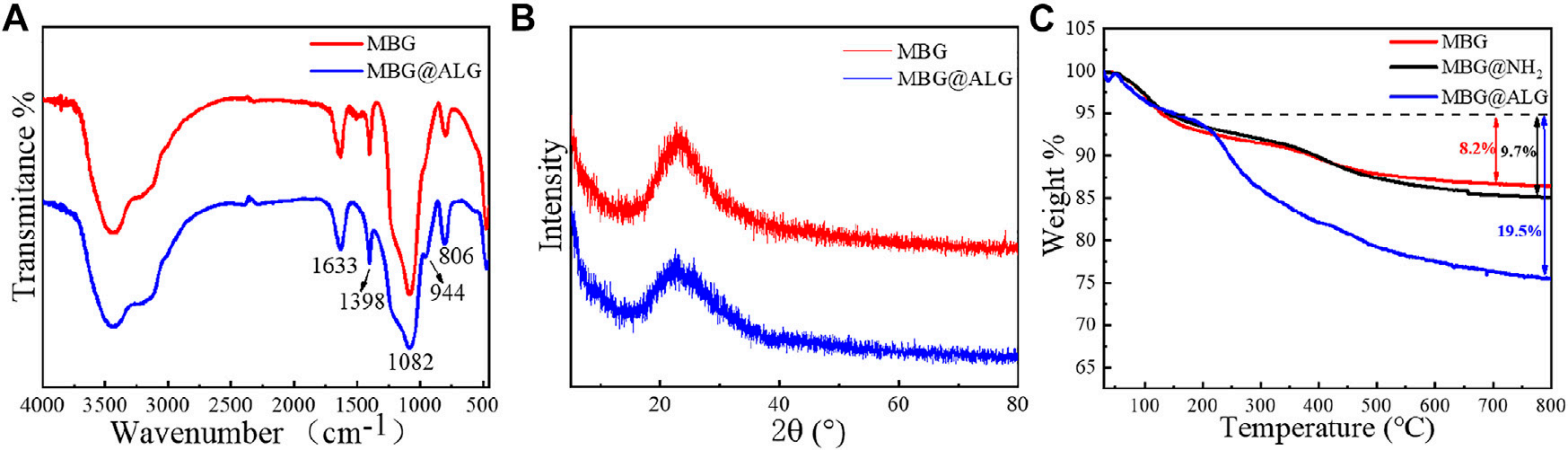
**(A)** FTIR spectra of MBG and MBG@ALG, **(B)** XRD patterns of MBG and MBG@ALG, and **(C)** TGA curve of MBG, MBG@NH_2_, and MBG@ALG.

In the FT-IR spectrum of MBG, the peaks at 1,080 cm^−1^, 806 cm^−1^, and 1,633 cm^−1^ (Figure 3A) were ascribed to Si–O–Si, O–Si–O, and Si–OH bands, respectively (Yan et al., 2017). For the spectrum of MBG@ALG, a new peak located at 944 cm^−1^ was corresponded to the C─O stretching, which was derived from ALG (Ding et al., 2021), demonstrating that ALG was successfully anchored onto MBG. The XRD patterns displays a wide-angle diffraction peak centered at 2θ = 20.4°, which was attributed to the amorphous nature of pristine MBG. After ALG was grafted, the wide-angle diffraction peak did not change, which is in consistence with the pattern of MBG (Figure 3B), demonstrating that the original crystalline structure of MBG remains unaltered after ALG surface modification. The TGA results showed that the weight loss of MBG@ALG was 24.7%, which consist of 5.2% weight loss of absorbed solvent before 150°C and 19.5% weight loss of the ALG between 150 and 800°C (Figure 3C). The 13.4% weight loss of MBG was due to the absorption of the solvent. The results show that the ALG coating was successfully prepared on the MBG surface.

**FIGURE 4:**
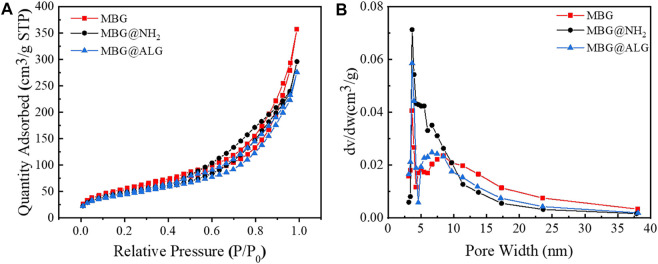
N_2_ adsorption–desorption isotherms **(A)** and pore-size distribution **(B)** of MBG, MBG@NH_2_, and MBG@ALG.

The N_2_ adsorption isotherms and pore size distribution of MBG, MBG@NH_2_, and MBG@ALG demonstrated the characteristic of mesoporous (Figure 4). The surface area, pore volume, and pore diameter of MBG were 196 m^2^/g, 0.55 cm^3^/g, and 11.3 nm (Table 1), respectively. After being treated with APTES, the surface area, pore volume, and pore diameter showed some changes, and the corresponding results of MBG@NH_2_ were found to be 171 m^2^/g, 0.458 cm^3^/g, and 10.7nm, respectively. When ALG polymer chains were grated with MBG@NH_2_, evident changes of the surface area, pore volume, and pore diameter of MBG@ALG were found, and the results were 162 m^2^/g, 0.427 cm^3^/g, and 10.5 nm (Table 1), respectively, much smaller than that of MBG, further demonstrating that ALG was successfully grafted on the surface and have an evident affection on its physical properties.

**TABLE 1:** Summary of the including specific surface area, pore volume, and pore size as determined from N_2_ adsorption–desorption isotherms.

Sample	Specific surface area (m^2^/g)	Pore volume (cm^3^/g)	Pore size (nm)
MBG	196	0.552	11.3
MBG@NH2	171	0.458	10.7
MBG@ALG	162	0.427	10.5

### 3.2 Drug loading and release assays

**FIGURE 5:**
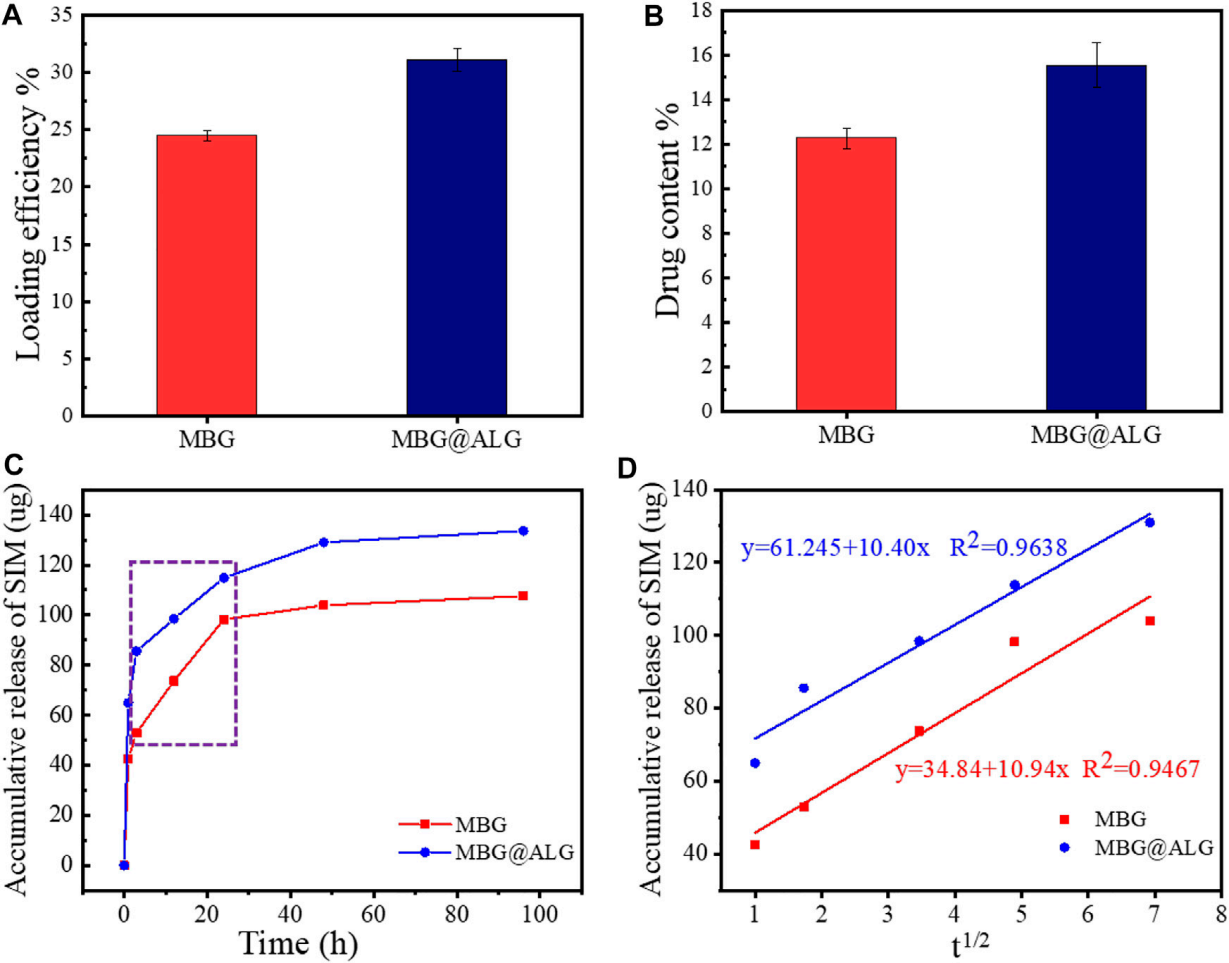
**(A)** Loading efficiency of MBG and MBG@ALG, **(B)** drug content of MBG and MBG@ALG, **(C)** release curves of MBG and MBG@ALG, and **(D)** the linear relationship between drug release and the square root of release time at the Higuchi model release phase.

The surface can significantly alter the drug loading efficiency and the drugs release time, and various surface molecules are found to be very useful for improving drug delivery (Cui et al., 2020). SIM was selected as a model drug, the loading efficiency of MBG was 24.5% (Xiao et al., 2019a; Xiao et al., 2019b). After ALG polymer chains were grated on the MBG, the loading efficiency was found to be improved, and the result was 31.2% (Figure 5A). The drug content of MBG and MBG@ALG was 12.2% and 15.6%, respectively (Figure 5B). The results demonstrated that ALG polymer chains can significantly improve the drug loading efficiency of MBG.

The drug release curves of MBG and MBG@ALG can be divided into three stages (Figure 5C). In the first stage, a burst release could be observed in the curves of MBG and MBG@ALG, which can be ascribed to the adsorbed SIM on the surface of MBG and MBG@ALG, rapidly diffusing into the solution. In the second stage, the release velocity of the SIM was significantly lower than that of the first stage, while this period of drug release is resulted from the SIM molecules which have entered the mesoporous channel, and therefore it takes a long time for the drug molecules to diffuse into the solution. However, when compared with the drug release curves in the second stage, it can be found that the release rate of SIM from MBG is faster than that of MBG@ALG. This is because the ALG polymer chains on the surface of MBG@ALG can hinder the diffusion of the SIM. In the third stage (after 48 h), the drug release rate decays to nearly zero, which means that, in this stage the drug diffusion and drug adsorption is dynamically balanced.

Furthermore, as shown in Figure 5C, in the second stage, there was an evident linear region both in the curves of MBG and MBG@ALG (shown in the rectangle), the drug release rate is linear with time, which is consistent with the zero-order kinetics (Marcos Luciano, 2015). In addition, on considering the porous structure of MBG and MBG@ALG, we try to use the Higuchi model to describe the release of SIM, the relationship between drug release amount and the square root of time was calculated and shown in Figure 5D, showing linear relationships and equations, implying that the release profiles can be explained *via* the Higuchi model, and this result is also consistent with the published work about MBG (Xiao et al., 2019a; Zhu and Stefan, 2019). All these results may demonstrate that ALG modification can improve the drug loading efficiency of MBG and prolong the drug release time without affecting its release mechanism.

### 3.3 *In vitro* bioactivity

**FIGURE 6:**
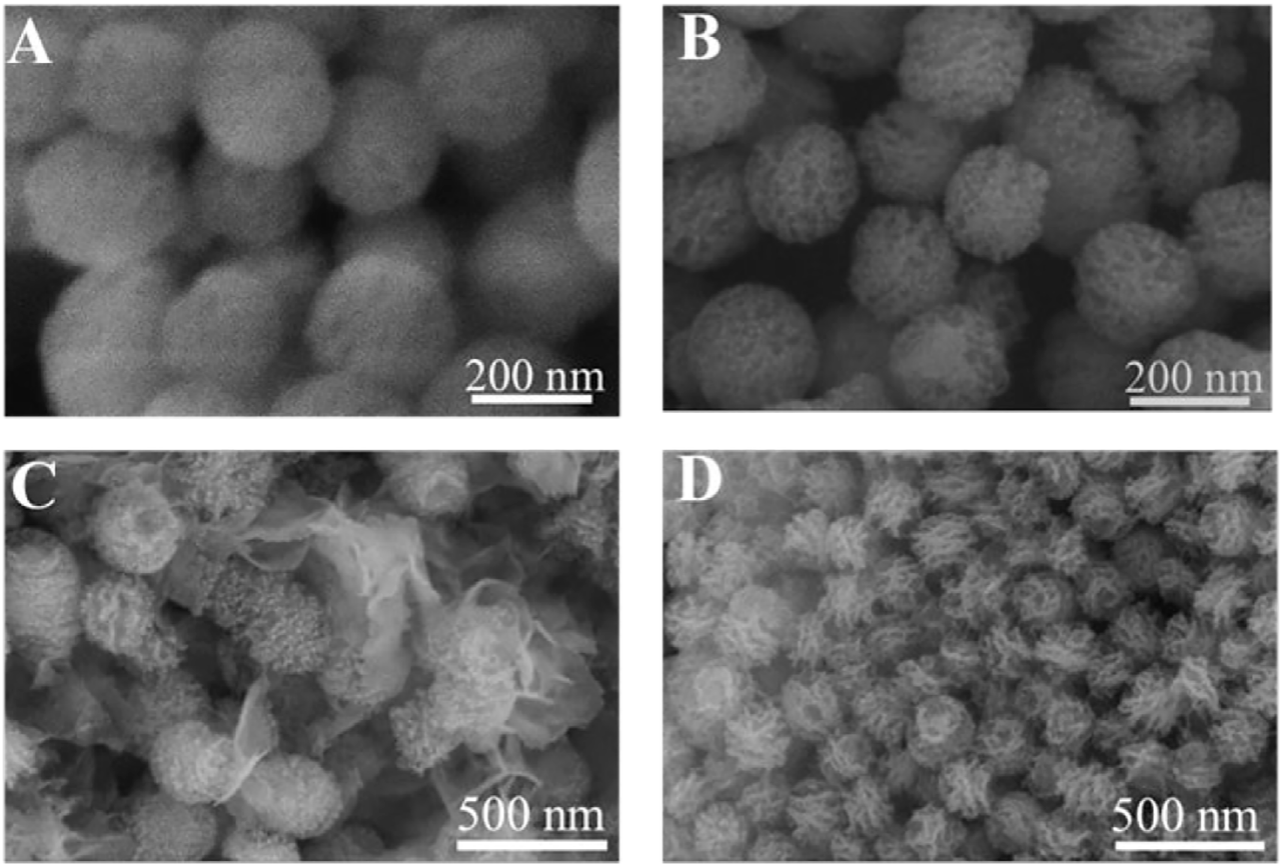
SEM image of MBG **(A)** and MBG@ALG **(B)**. SEM image of MBG **(C)** and MBG@ALG **(D)** after treated with SBF for 7 days.

The surface ligands have great influence on mineral deposition, such as defining the mineral-deposition site and affecting the morphology of apatite (Meldrum F, 2008; Gordon and Joester, 2011). The SEM images showed both MBG and MBG@ALG are spherical nanoparticles with a particle size of about 200 nm (Figures 6A,B). After treated in SBF, many irregular apatite plates are formed around MBG (Figure 6C), which may be that the released Ca^2+^ increases the concentration of Ca^2+^ around MBG, inducing nucleation and the growth of apatite. Generally, due to the biodegradation ability of MBG, when the MBG samples were immersed into the SBF solution, the mineralization behaviors also happens and this may be a dissolve-regrowth scheme. After the ALG polymer chains were grafted on MBG, compared with MBG that treated with SBF for 7 days, a regular and tightly arranged plate structure can be observed on the surface of the MBG@ALG (Figure 6D). Since SBF were used as a mineralization medium, the ALG can chelate Ca^2+^ from the solution and induce nucleation as crystal seed, therefore, orderly growth of apatite on the surface of MBG@ALG formed, in addition, the amount of plate apatite of MBG@ALG (Figure 6D) is much more than that of MBG (Figure 6C), demonstrating that grafting of ALG can control the morphology and accelerate the mineralization of apatite, which may broaden the application of MBG in bone tissue engineering.

### 3.4 Biocompatibility test of MBG and MBG@ALG

**FIGURE 7:**
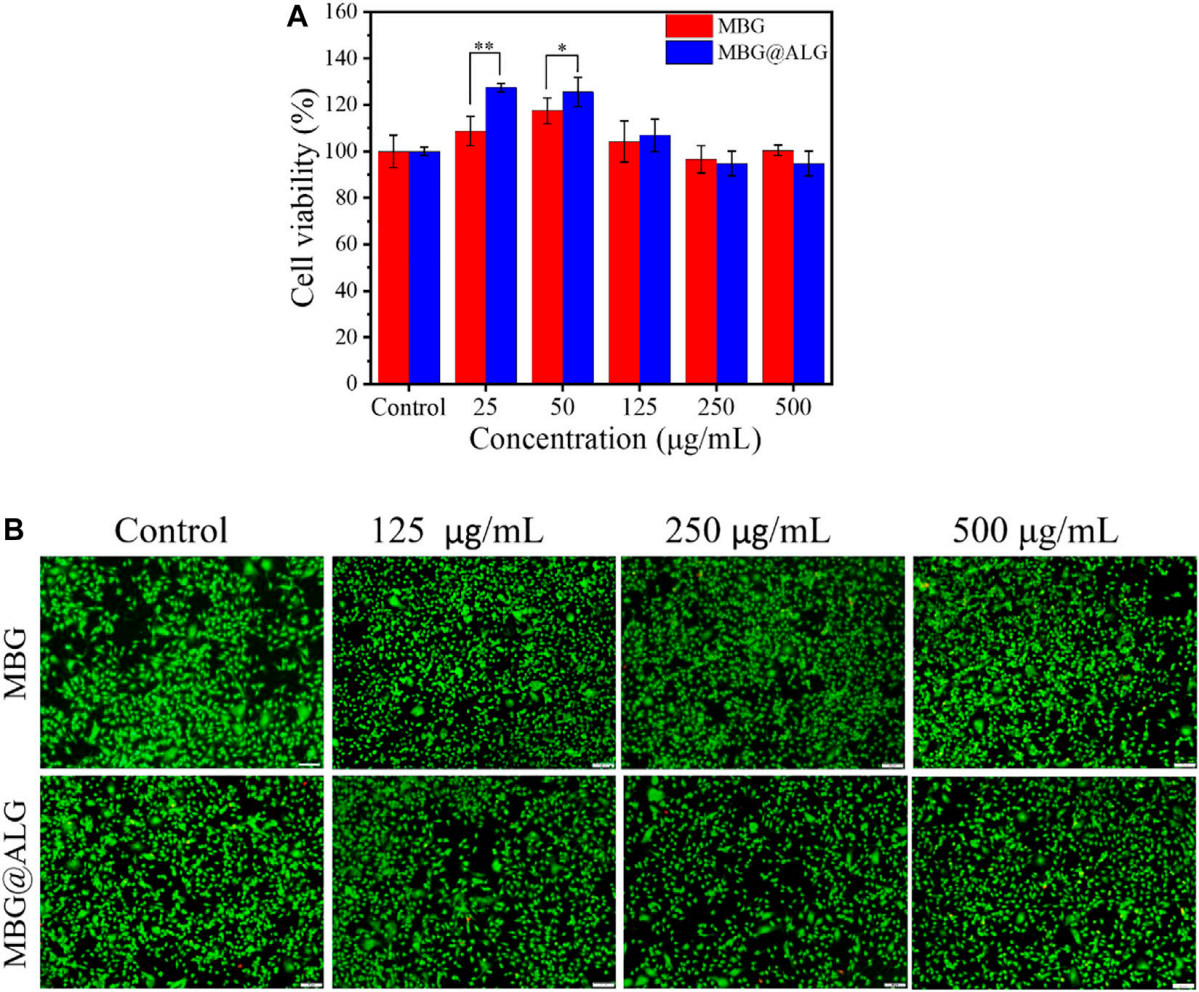
**(A)** Cell viability of MC3T3-E1 cells after being cocultured with MBG and MBG@ALG. **(B)** AO/EB staining fluorescence images of MC3T3-E1 cells after being cocultured with MBG and MBG@ALG. (Bar = 100 μm n = 5, *p < 0.05, **p < 0.01)

MBG and MBG@ALG show excellent biocompatibility, even when the concentration of the materials reached to 500 μg/ml. When the concentration was 25 μg/ml, the cell viability of MBG and MBG@ALG was 108% and 127%, respectively. At the concentration of 50 μg/ml, the cell viability of MBG and MBG@ALG was 117% and 125% (Figure 7A), respectively. These indicate that MBG and MBG@ALG can significantly promote cell proliferation at low concentrations, and the cell viability of MBG@ALG is significantly higher than that of MBG at the same concentration. When the concentration of MBG was 100 , 250 , and 500 μg/ml, the cell viability was 104%, 96%, and 100%, respectively. When the concentration of MBG@ALG was 100 , 250 , and 500 μg/ml, the cell viability was 107%, 94%, and 95%, respectively (Figure 7A). MBG and MBG@ALG showed good compatibility at these concentrations, and the effects of MBG and MBG@ALG on cell viability at these concentrations were not significantly different.

AO/EB staining further verified the excellent biocompatibility of MBG@ALG (Figure 7B). The cells survived well after being cocultured with the material (green fluorescence), with only a few apoptotic cells (red fluorescence). These results of MBG@ALG was biocompatible and can promote cell proliferation at low concentrations.

### 3.5 Osteogenesis differentiation of MC3T3-E1 cells

**FIGURE 8:**
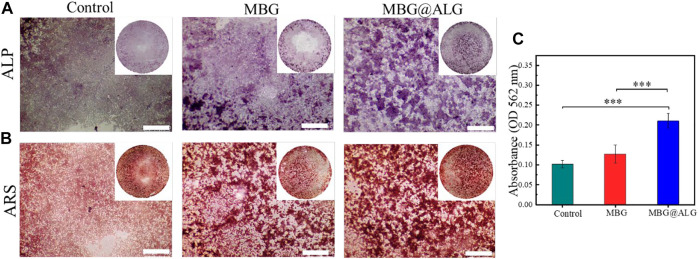
**(A)** ALP staining after MC3T3-E1 cell was cocultured with MBG and MBG@ALG for 7 days. **(B) **ARS staining after MC3T3-E1 cell was cocultured with MBG and MBG@ALG for 14 days. **(C)** ARS staining quantification after 14 days. (Bar = 500 μm, ***p < 0.001)

MBG has good osteoconductivity and osteoinductive properties, which can promote the osteogenic differentiation of cells. ALP, an early marker of osteogenic differentiation and mineralized nodules-a late marker of osteogenic differentiation, were detected by ALP staining and ARS staining, respectively. After the cells were cocultured with the MBG and MBG@ALG for 7 days, respectively, the purple color in the MBG@ALG group was significantly stronger than that in the MBG group (Figure 8A), indicating that the cells in the MBG@ALG group expressed more ALP. This result demonstrated that MBG@ALG has a great effect on promoting the early osteogenic differentiation of MC3T3-E1 cells. After 14 days of coculture, the ARS staining images and quantification date showed that the number and density of mineralized nodules in the MBG@ALG group were higher than those in the MBG group (Figures 8B,C), indicating that MBG@ALG had an excellent effect on promoting extracellular matrix mineralization. These results demonstrate that the surface modification of ALG promotes both early osteogenic differentiation and late osteoblast maturation, which will further broaden the application of MBG.

## 4 Conclusion

In this work, an ALG-modified MBG was successfully prepared (MBG@ALG), while the ALG chains on the surface of MBG could improve the drug loading efficiency and restrain the drug release speed. Furthermore, MBG@ALG showed excellent biocompatibility and could promote the osteogenic differentiation of cells, demonstrating that the surface functionalization of MBG with ALG has a significant impact on its properties, which may promote the further application of MBG in drug delivery and bone tissue engineering.

## Data Availability

The raw data supporting the conclusion of this article will be made available by the authors, without undue reservation.

## References

[B1] Ahmad Raus R. Wan Nawawi W. M. F. Nasaruddin R. R. (2021). Alginate and alginate composites for biomedical applications. Asian J. Pharm. Sci. 16, 280–306. 10.1016/j.ajps.2020.10.001 34276819 PMC8261255

[B2] Bae J. Son W. S. Yoo K. H. Yoon S. Y. Bae M. K. Lee D. J. (2019). Effects of poly(amidoamine) dendrimer-coated mesoporous bioactive glass nanoparticles on dentin remineralization. Nanomaterials 9, 591. 10.3390/nano9040591 PMC6523905 30974829

[B3] Boles M. A. Ling D. Hyeon T. Talapin D. V. (2016). Erratum: The surface science of nanocrystals. Nat. Mat. 15, 364. 10.1038/nmat4578 26906962

[B4] Bygd H. C. Bratlie K. M. (2016). The effect of chemically modified alginates on macrophage phenotype and biomolecule transport. J. Biomed. Mat. Res. A 104, 1707–1719. 10.1002/jbm.a.35700 26939998

[B5] Cao C. Hong C. Li Y. Li G. Jiang G. (2020). A long‐term and stable surface modification method for lanthanide doped upconversion nanoparticles by oxidized alginate. Z. Anorg. Allg. Chem. 646, 1607–1610. 10.1002/zaac.202000262

[B6] Cui L. Zhang J. Zou J. Yang X. Guo H. Tian H. (2020). Electroactive composite scaffold with locally expressed osteoinductive factor for synergistic bone repair upon electrical stimulation. Biomaterials 230, 119617. 10.1016/j.biomaterials.2019.119617 31771859

[B7] Das M. P. Pandey G. Neppolian B. Das J. (2021). Design of poly-l-glutamic acid embedded mesoporous bioactive glass nanospheres for pH-stimulated chemotherapeutic drug delivery and antibacterial susceptibility. Colloids Surfaces B Biointerfaces 202, 111700. 10.1016/j.colsurfb.2021.111700 33756297

[B8] Ding X. Shi J. Wei J. Li Y. Wu X. Zhang Y. (2021). A biopolymer hydrogel electrostatically reinforced by amino-functionalized bioactive glass for accelerated bone regeneration. Sci. Adv. 7, eabj7857. 10.1126/sciadv.abj7857 34890238 PMC8664252

[B9] Gordon L. M. Joester D. (2011). Nanoscale chemical tomography of buried organic-inorganic interfaces in the chiton tooth. Nature 469, 194–197. 10.1038/nature09686 21228873

[B10] Heinz H. Pramanik C. Heinz O. Ding Y. Mishra R. K. Marchon D. (2017). Nanoparticle decoration with surfactants: Molecular interactions, assembly, and applications. Surf. Sci. Rep. 72, 1–58. 10.1016/j.surfrep.2017.02.001

[B11] Kango S. Kalia S. Celli A. Njuguna J. Habibi Y. Kumar R. (2013). Surface modification of inorganic nanoparticles for development of organic–inorganic nanocomposites—a review. Prog. Polym. Sci. 38, 1232–1261. 10.1016/j.progpolymsci.2013.02.003

[B12] Kargozar S. Kermani F. Mollazadeh Beidokhti S. Hamzehlou S. Verné E. Ferraris S. (2019). Functionalization and surface modifications of bioactive glasses (BGs): Tailoring of the biological response working on the outermost surface layer. Materials 12, 3696. 10.3390/ma12223696 PMC6888252 31717516

[B13] Kerschenmeyer A. Arlov. Ø. Malheiro V. Steinwachsd M. Rottmar M. Maniura-Weber K. (2017). Anti-oxidant and immune-modulatory properties of sulfated alginate derivatives on human chondrocytes and macrophages. Biomater. Sci. 00, 1756–1765. 10.1039/c7bm00341b 28643827

[B14] Kim T. H. Singh R. K. Kang M. S. Kim J. H. Kim H. W. (2016). Gene delivery nanocarriers of bioactive glass with unique potential to load BMP2 plasmid DNA and to internalize into mesenchymal stem cells for osteogenesis and bone regeneration. Nanoscale 8, 8300–8311. 10.1039/c5nr07933k 27035682

[B15] Kodiyan A. Silva E. A. Kim J. Aizenberg M. Mooney D J. (2012). Surface modification with alginate-derived polymers for stable, protein-repellent, long-circulating gold nanoparticles. Acs Nano 6, 4796–4805. 10.1021/nn205073n 22650310

[B16] Kokubo T. Takadama H. (2006). How useful is SBF in predicting *in vivo* bone bioactivity? Biomaterials 27, 2907–2915. 10.1016/j.biomaterials.2006.01.017 16448693

[B17] Lee K. Y. Moone y. D. J. (2012). Alginate: Properties and biomedical applications. Prog. Polym. Sci. 37, 106–126. 10.1016/j.progpolymsci.2011.06.003 22125349 PMC3223967

[B18] Lopez-Noriega A. Arcos D. Vallet-Regi M. (2010). Functionalizing mesoporous bioglasses for long-term anti-osteoporotic drug delivery. Chem. Eur. J. 16, 10879–10886. 10.1002/chem.201000137 20661959

[B19] Marcos Luciano B. (2015). Strategies to modify the drug release from pharmaceutical systems. Sawston, United Kingdom: Woodhead Publishing, 63–86.

[B20] Meldrum F C. (2008). Controlling mineral morphologies and structures in biological and synthetic systems. Chem. Rev. 108, 4332–4432. 10.1021/cr8002856 19006397

[B21] Pontremoli C. Izquierdo-Barba I. Montalbano G. Vallet-Regi M. Vitale-Brovarone C. Fiorilli S. (2020). Strontium-releasing mesoporous bioactive glasses with anti-adhesive zwitterionic surface as advanced biomaterials for bone tissue regeneration. J. colloid interface Sci. 563, 92–103. 10.1016/j.jcis.2019.12.047 31869588 PMC7116262

[B22] Rahaman M. N. Day D. E. Bal B. S. Fu Q. Jung S. B. Bonewald L. F. (2011). Bioactive glass in tissue engineering. Acta biomater. 7, 2355–2373. 10.1016/j.actbio.2011.03.016 21421084 PMC3085647

[B23] Ravanbakhsh M. Labbaf S. Karimzadeh F. Pinna A. Houreh A. B. Nasr-Esfahani M. H. (2019). Mesoporous bioactive glasses for the combined application of osteosarcoma treatment and bone regeneration. Mater. Sci. Eng. C 104, 109994. 10.1016/j.msec.2019.109994 31500021

[B24] Schumacher M. Habibovic P. van Rijt S. (2021). Mesoporous bioactive glass composition effects on degradation and bioactivity. Bioact. Mater. 6, 1921–1931. 10.1016/j.bioactmat.2020.12.007 33385099 PMC7758280

[B25] Schumacher M. Reither L. Thomas J. Kampschulte M. Gbureck U. Lode A. (2017). Calcium phosphate bone cement/mesoporous bioactive glass composites for controlled growth factor delivery. Biomater. Sci. 5, 578–588. 10.1039/c6bm00903d 28154869

[B26] Sharifi E. Bigham A. Yousefiasl S. Trovato M. Ghomi M. Esmaeili Y. (2022). Mesoporous bioactive glasses in cancer diagnosis and therapy: Stimuli-responsive, toxicity, immunogenicity, and clinical translation. Adv. Sci. 9, e2102678. 10.1002/advs.202102678 PMC8805580 34796680

[B27] Vallet-Regi M. Salinas A. J. (2021). Mesoporous bioactive glasses for regenerative medicine. Mater. today Bio 11, 100121. 10.1016/j.mtbio.2021.100121 PMC8327654 34377972

[B28] Wang X. Zeng D. Weng W. Huang Q. Zhang X. Wen J. (2018). Alendronate delivery on amino modified mesoporous bioactive glass scaffolds to enhance bone regeneration in osteoporosis rats. Artif. cells, nanomedicine, Biotechnol. 46, 171–181. 10.1080/21691401.2018.1453825 29688044

[B29] Xiao J. Wan Y. Yang Z. Huang Y. Zhu Y. Yao F. (2019). Bioactive glass nanotube scaffold with well-ordered mesoporous structure for improved bioactivity and controlled drug delivery. J. Mater. Sci. Technol. 35, 1959–1965. 10.1016/j.jmst.2019.04.027

[B30] Xiao J. Wan Y. Yang Z. Huang Y. Zhu Y. Yao F. (2019). Simvastatin-loaded nanotubular mesoporous bioactive glass scaffolds for bone tissue engineering. Microporous Mesoporous Mater. 288, 109570. 10.1016/j.micromeso.2019.109570

[B31] Xu Y. Wu P. Feng P. Guo W. Yang W. Shuai C. (2018). Interfacial reinforcement in a poly-l-lactic acid/mesoporous bioactive glass scaffold via polydopamine. Colloids Surfaces B Biointerfaces 170, 45–53. 10.1016/j.colsurfb.2018.05.065 29870952

[B32] Yan H. Chen X. Bao C. Wu S. He S. Lin Q. (2019). Alginate derivative-functionalized silica nanoparticles: Surface modification and characterization. Polym. Bull. Berl. 77, 73–84. 10.1007/s00289-019-02736-9

[B33] Yan S. Sun Y. Chen A. Liu L. Zhang K. Li G. (2017). Templated fabrication of pH-responsive poly(l-glutamic acid) based nanogels via surface-grafting and macromolecular crosslinking. RSC Adv. 7, 14888–14901. 10.1039/c7ra00631d

[B34] Yao H. Zhu M. Wang P. Liu Y. Wei J. (2021). Combination of mussel inspired method and “thiol-michael” click reaction for biocompatible alginate-modified carbon nanotubes. Nanomaterials 11, 2191. 10.3390/nano11092191 34578507 PMC8471357

[B35] Zhao D. Zhu T. Li J. Cui L. Zhang Z. Zhuang X. (2021). Poly(lactic-co-glycolic acid)-based composite bone-substitute materials. Bioact. Mater. 6, 346–360. 10.1016/j.bioactmat.2020.08.016 32954053 PMC7475521

[B36] Zhu Y. Stefan K. (2019). Comparison of the *in vitro* bioactivity and drug release property of mesoporous bioactive glasses (MBGs) and bioactive glasses (BGs) scaffolds. Microporous Mesoporous Mater. 118, 176–182. 10.1016/j.micromeso.2008.08.046

